# Diagnosis and Treatment of Neonatal Diabetes Caused by ATP-Channel Mutations: Genetic Insights, Sulfonylurea Therapy, and Future Directions

**DOI:** 10.3390/children12020219

**Published:** 2025-02-12

**Authors:** Michela Trada, Chiara Novara, Martina Moretto, Edoardo Burzi, Davide Tinti, Luisa De Sanctis

**Affiliations:** 1 Department of Pediatric Endocrinology, Regina Margherita Children’s Hospital, 10126 Torino, Italy; mtrada@cittadellasalute.to.it (M.T.); luisa.desanctis@unito.it (L.D.S.); 2 Department of Public Health and Pediatric Sciences, University of Torino, 10126 Torino, Italy

**Keywords:** neonatal diabetes, KCNJ11, ABCC8, sulfonylureas, genetic mutations, ATP-sensitive potassium channels, genetic testing, personalized treatment, neonatal diabetes management, DEND syndrome, sulfonylurea therapy, CRISPR, prenatal diagnosis

## Abstract

Background: Neonatal diabetes (NDM) is a rare genetic disorder diagnosed in infants under six months of age, characterized by persistent hyperglycemia resulting from insufficient or absent insulin production. Unlike the more common forms of diabetes, such as type 1 diabetes (T1D) and type 2 diabetes (T2D), NDM is predominantly caused by monogenic mutations affecting ATP-sensitive potassium (K-ATP) channels in pancreatic beta cells. The most common mutations involved in NDM are found in the KCNJ11 and ABCC8 genes, which encode the Kir6.2 and SUR1 subunits of the K-ATP channel, respectively. These mutations prevent normal insulin secretion by disrupting the function of the K-ATP channel. While genetic advances have identified about 40 genes implicated in NDM, the KCNJ11 and ABCC8 mutations are most commonly seen. Methods: This review provides a comprehensive exploration of the genetic basis, clinical presentation, and treatment strategies for NDM including the role of sulfonylureas, which have revolutionized the management of this condition. Furthermore, it presents a detailed case study of an infant diagnosed with an ABCC8 mutation, illustrating the pivotal role of genetic testing in guiding clinical decisions. Conclusions: Finally, the article discusses challenges in management, such as the persistence of neurological impairments, and outlines potential directions for future research including genetic therapies and prenatal diagnosis.

## 1. Introduction

Neonatal diabetes (NDM) is a rare but increasingly recognized form of diabetes, typically diagnosed in infants within the first six months of life. Unlike type 1 diabetes (T1D) and type 2 diabetes (T2D), NDM, in most cases, is caused by genetic mutations that impair insulin secretion due to defects in the ATP-sensitive potassium channels (K-ATP) of pancreatic beta cells [1]. These channels are critical for regulating insulin secretion in response to blood glucose levels, and mutations in the genes encoding these channels lead to chronic hyperglycemia [2]. The condition is relatively rare, with an incidence of approximately 1 in 90,000 to 160,000 live births globally, though this can vary depending on the population. In regions with higher consanguinity rates, such as northwest Saudi Arabia, the prevalence can increase to as high as 1 in 20,000 [2]. Despite its rarity, NDM is a serious health condition that requires early diagnosis and personalized treatment strategies. Genetic testing is essential to confirm the diagnosis and identify the specific genetic mutation, which plays a crucial role in guiding treatment choices such as the transition from insulin therapy to oral sulfonylureas [3].

Understanding the genetic mechanisms underlying NDM has provided insights into the pathophysiology of the disease and has led to the development of more targeted therapies. KCNJ11 and ABCC8 are the most commonly implicated genes, and mutations in these genes affect the Kir6.2 and SUR1 subunits of the K-ATP channels. These mutations can lead to both transient and permanent forms of NDM, depending on the nature of the mutation [4]. The term transient diabetes refers to a condition that usually tends to resolve in childhood (usually by 13–18 weeks of age) but may recur later in life. Permanent diabetes affects a percentage of patients who will require lifelong treatment with insulin [5]. This review will explore these genetic mechanisms, the clinical implications of NDM, the therapeutic advances that have improved patient outcomes, and future directions in the field [5,6].

## 2. Genetic Mechanisms of Neonatal Diabetes

NDM is primarily caused by gain-of-function mutations in the KCNJ11 and ABCC8 genes. These mutations lead to a malfunctioning K-ATP channel that remains open despite elevated glucose levels in the blood, resulting in impaired insulin secretion. The KCNJ11 gene encodes Kir6.2, a subunit of the K-ATP channel, while the ABCC8 gene encodes the SUR1 subunit. The KCNJ11 gene is located on chromosome 11 and contains 4 exons, while the ABCC8 gene, also located on chromosome 11, has 39 exons, with an alternatively spliced exon 17 [1]. Mutations in ABCC8 can cause variable effects, with transient or permanent NDM developing based on how much the mutation alters ATP sensitivity.

These mutations affect the ATP sensitivity of the channel, making it less responsive to ATP, which is required to close the channel. This results in persistent hyperglycemia, as insulin is not adequately secreted in response to blood glucose levels. The mutations in KCNJ11 tend to lead to permanent neonatal diabetes, whereas mutations in ABCC8 can result in either transient or permanent forms of NDM. Transient NDM (TNDM) often resolves in early childhood, while permanent NDM (PNDM) persists lifelong, depending on ATP sensitivity alteration caused by ABCC8 mutations [2].

In addition to affecting insulin secretion, K-ATP channels are also expressed in other tissues including neurons. This is why many patients with KCNJ11 and ABCC8 mutations experience neurological symptoms such as developmental delay, epilepsy, hypotonia, and autism. These symptoms, collectively known as DEND syndrome (developmental delay, epilepsy, and neonatal diabetes), occur most frequently in patients with mutations that drastically reduce the ATP sensitivity of the channel. This condition exemplifies how mutations in a single gene can have both metabolic and neurological consequences [3].

## 3. Clinical Presentation and First Approach to NDM

The clinical presentation of ND is usually seen in the first six months of life. Common symptoms include polyuria, polydipsia, and failure to thrive. Infants may also present with unexplained weight loss, and in severe cases, diabetic ketoacidosis (DKA) can occur. DKA is characterized by metabolic acidosis, hyperglycemia, and ketonuria, which can often be the first clue of ND. Given that the symptoms of ND overlap with those of infections, it is frequently misdiagnosed as sepsis in neonates.

We report here a paradigmatic case that describes diagnostic, management, and therapeutic challenges of ND: E. is a two-and-a-half-month-old baby, born at full term of a physiological pregnancy. He is the first-born child of healthy non-blood relatives. Birth weight: 2990 g (appropriate for gestational age, APGAR 9/9, 1 point deducted for mild respiratory adjustment).

He was taken to the emergency room of a suburban hospital for a protracted state of discomfort over several days, characterized by inconsolable crying in the absence of other symptoms. An abdominal ultrasound was performed, which revealed abundant meteorism, with no possibility of ruling out intestinal invagination.

Given the worsening clinical condition (hyporeactivity and skin marbling), a blood draw was performed for blood culture, and empirical antibiotic therapy (ceftriaxone) was started on suspicion of septic shock. Contextually, a capillary blood gas test was conducted that revealed severe metabolic acidosis (pH 7.026, pCO_2_ 18.6 mmHg, HCO_3_ 4.8 mmol/L, base excess −26.1 mmol/L) and severe hyperglycemia (980 mg/dL), therefore, intravenous hydration with normal saline was immediately initiated. The infant was transferred to the spoke center Regina Margherita Children’s Hospital Surgical Emergency Department for further diagnosis and continuation of care.

At admission, E. was in poor general condition, with a distressed appearance and inconsolable crying. He was tachycardic and tachypneic (respiratory rate 60/min) with good oxygen saturations in room air. The pattern of severe metabolic acidosis and hyperglycemia was confirmed on ABG (arterial blood gas); elevated ketonemia and ketonuria confirmed the suspicion of diabetic ketoacidosis (DKA).

The national board-approved DKA protocol was applied: intravenous hydration with potassium-corrected saline and intravenous insulin infusion (Humulin R insulin at a rate of 0.15 U/h) was commenced. Hence, E. was transferred to the intensive care unit, where he was started on respiratory support with high-flow oxygen therapy.

In the following days, there was an improvement in the clinical conditions and the blood gas parameters, along with good glycemic control. However, due to persistent irritability and following an episode of convergent squinting, a brain MRI was performed that showed subacute cerebral venous sinus thrombosis sectors. E. was then started on subcutaneous anticoagulant therapy with heparin that was discontinued after six weeks. After only ten days, a follow-up MRI showed a resolution of the thrombosis.

After six days of intravenous insulin therapy, subcutaneous administration was started using a Medtronic 780G pump (Milan, Italy) integrated with continuous glycemic monitoring with a Guardian 4 sensor (Medtronic, Milan, Italy). Due to his minimal insulin requirements and frequent and numerous dairy meals, it was difficult to adjust the amounts of insulin to achieve reasonable glycemic compensation. At discharge, the patient had a daily insulin dose of 2.7 IU, divided into 0.7 IU basal and 2 IU bolus (equal to 0.4 IU/kg/day). To exclude morphologic abnormalities of the pancreas (which can be present in some forms of ND), an ultrasound and an abdominal MRI were performed during the hospitalization, which did not show any abnormality. In addition, the study of pancreatic exocrine function (fecal elastase) was also within the limits.

Given the young age of onset of diabetes of probable genetic etiology, an NGS analysis of a panel of genes associated with monogenic diabetes was performed, which identified the c.638T>C [p.(Leu2132Pro)] variant in the ABCC8 gene, classified as probably pathogenic. Activating mutations in ABCC8, which encodes for the outer subunit of the ATP-sensitive potassium channel (K-ATP channel), result in the inability to release insulin, leading to a form of monogenic diabetes with autosomal dominant transmission. The K-ATP channel is the pharmacological target of sulfonylurea, which acts by promoting channel closure and subsequent insulin release. This drug is a valid and effective therapeutic alternative to insulin in these forms of ND.

Three weeks later, an admission was scheduled to test the therapeutic switch from subcutaneous insulin to oral sulfonylurea. Glibenclamide was started at a dosage of 0.2 mg/kg in three daily administrations, with progressive increases according to glycemic values and concomitant reduction in insulin delivery until total discontinuation (after three days of the start of oral therapy). Sulfonylurea therapy was introduced on Day 21, and insulin was completely discontinued by Day 24. In support of the efficacy of therapy, the C-peptide value (the equivalent of insulin secretion) also increased from an initial value of 0.2 μg/L to a value of 3.0 μg/L. The patient was discharged with only oral therapy (glibenclamide 0.5 mg/kg/day) and a continuous glycemic monitoring sensor. E. continued follow-up at our center, and given the goodness of the glycemic values, the therapy with glibenclamide was gradually scaled up until its complete discontinuation after approximately four and a half months. At about four months of age, weaning was started, which had always been characterized by a large caloric meal intake, which the infant tolerated well. Moreover, for the finding of hypertransaminasemia (AST 135 U/L, ALT 215 U/L), E. also underwent gastroenterological evaluation, and therefore, several instrumental examinations (such as abdominal ultrasound and Fibroscan) and blood tests to rule out infectious or immune causes of hepatopathy. All of these in-depth examinations were found to be normal. Post-treatment monitoring showed a significant improvement in neurodevelopmental milestones within the following months.

To confirm the diagnosis, genetic testing is essential. It helps identify specific mutations in the KCNJ11 and ABCC8 genes, which is crucial for guiding treatment decisions. Next-generation sequencing (NGS) is the preferred method for identifying these mutations, as it can provide accurate and comprehensive results [2]. As recommended in the latest ISPAD guidelines, genetic testing is recommended in patients diagnosed with ND under the age of 6 months, with the aim of identifying the genetic etiology, which could subsequently influence the treatment of ND. Patients carrying different mutations require different treatments, which may include insulin, oral antidiabetic agents, or even, rarely, bone marrow transplantation [7,8].

## 4. Sulfonylurea Therapy: A Breakthrough in NDM Management

The discovery that sulphonylureas can be used to treat neonatal diabetes (NDM) caused by mutations in the KCNJ11 and ABCC8 genes represented a major breakthrough in the management of this rare and difficult condition. In particular, these were introduced into clinical practice for NDM from 2004 to 2006 [9]. Sulfonylureas work by binding to the SUR1 subunit of the K-ATP channel, promoting its closure and stimulating insulin secretion from pancreatic beta cells. This mechanism of action is particularly beneficial for patients with mutations in the KCNJ11 and ABCC8 genes, as these mutations lead to K-ATP channels that are less sensitive to ATP, causing them to remain open even in the presence of elevated glucose levels [10], Figure S1.

Studies have indicated that up to 90% of patients with KCNJ11 mutations and 80–90% of those with ABCC8 mutations can successfully transition from insulin therapy to oral sulfonylureas [8]. This transition has been associated with improved glycemic control, reduced insulin requirements, and enhanced overall quality of life. The early introduction of sulfonylureas could mitigate neurodevelopmental delays associated with ATP-channel mutations. Importantly, sulfonylureas offer a more convenient and less invasive treatment option compared with insulin injections, which is especially beneficial for neonates and infants who may have difficulty adhering to frequent insulin injections [11].

However, despite the widespread success of this therapy, there are important caveats and challenges to consider. The effectiveness of sulfonylureas can vary depending on the specific mutation and the extent of the K-ATP channel dysfunction. Some mutations may cause the K-ATP channels to remain open for more extended periods, reducing the response to sulfonylureas. In these cases, a combination of sulfonylureas and insulin therapy may be required to achieve optimal glycemic control [12].

Mutations such as KCNJ11 G334D can reduce responsiveness to sulfonylureas, necessitating the careful monitoring of blood glucose levels and the adjustment of therapy accordingly [13]. This variability underscores the importance of personalized medicine, where treatment is tailored to the patient’s specific genetic mutation, clinical presentation, and response to therapy. Genetic testing is crucial in determining the appropriate treatment plan, ensuring that patients receive the most effective therapy for their particular mutation [14].

## 5. Challenges in NDM Management

Despite the substantial progress made in the management of NDM with sulfonylurea therapy, several challenges remain. One of the primary concerns is the neurological sequelae observed in many patients, particularly those with more severe mutations in the KCNJ11 and ABCC8 genes. These patients may experience developmental delays, learning disabilities, autism, and epilepsy, even after achieving good glycemic control [10].

These neurological complications are thought to be related to the expression of K-ATP channels in neurons, where defects in channel function may alter neuronal excitability. Studies have shown that these neurological symptoms are more pronounced in patients with severe mutations that drastically reduce the ATP sensitivity of the K-ATP channels [15]. While sulfonylureas can ameliorate the metabolic aspects of NDM, they do not necessarily prevent or treat neurological symptoms, which persist in many patients despite excellent glycemic control.

There is a growing body of research investigating the long-term effects of sulfonylureas on neurodevelopment. Although sulfonylureas are effective in normalizing glucose levels, the therapy may not fully reverse the underlying neuronal dysfunction caused by K-ATP channel mutations [11]. As a result, neuroprotective strategies are being explored to improve the long-term neurological outcomes of NDM patients. These may include neurotrophic factors, antioxidants, and other compounds that can promote neuronal health and function [12].

## 6. Prenatal Diagnosis and Early Intervention

Another promising area of research is the development of prenatal diagnosis techniques for NDM. Non-invasive prenatal testing (NIPT) has advanced significantly in recent years, and it is now possible to detect KCNJ11 and ABCC8 mutations early in pregnancy [12]. This has opened the possibility of prenatal therapy with sulfonylureas, which could improve outcomes for both the mother and fetus. However, there are potential risks associated with sulfonylurea use during pregnancy including fetal macrosomia and neonatal hypoglycemia. These complications arise due to excessive insulin secretion in response to sulfonylurea exposure, which can lead to overgrowth of the fetus and low blood glucose levels after birth [16].

Therefore, to prevent these adverse effects, careful monitoring is required during prenatal treatment with sulfonylureas. The potential for early intervention in prenatal NDM offers an exciting opportunity for improving the outcomes of affected pregnancies. However, more research is needed to determine the optimal timing and dosage of sulfonylurea therapy during pregnancy and to better understand its long-term effects on both the mother and child [17].

## 7. Genetic Therapies: The Future of NDM Treatment

Looking ahead, genetic therapies have considerable potential to provide curative treatments for NDM patients. CRISPR/Cas9 genome editing is a revolutionary technology that allows for precise genome editing, offering the possibility of correcting KCNJ11 and ABCC8 mutations at the DNA level. While this approach is still in its early stages, research is ongoing to determine whether CRISPR/Cas9 can be used to correct genetic mutations in patients with NDM, potentially offering a permanent cure for the disease [14].

In addition to CRISPR/Cas9, other emerging technologies, such as gene therapy and RNA-based therapies, may also play a role in the future treatment of NDM. These therapies could provide a long-lasting solution to the metabolic and neurological challenges posed by K-ATP channel dysfunction. However, much work remains before these therapies become available for clinical use. Clinical trials and further research will be necessary to determine their safety, efficacy, and potential impact on patient outcomes [16,17]. While CRISPR/Cas9 has garnered attention due to its versatility and efficiency, it is essential to evaluate the advantages and disadvantages of different gene editing techniques. For example, base editing allows for single-nucleotide changes without inducing double-strand breaks, reducing the risk of off-target effects. Prime editing, another emerging technique, provides greater precision and the ability to correct a wide variety of mutations. These alternatives may offer safer and more targeted approaches for treating neonatal diabetes (ND) in the future.

The ethical implications of gene editing in humans must also be carefully considered. While genetic therapies hold immense promise, they raise significant concerns regarding the long-term safety, potential unintended consequences, and accessibility. The use of gene editing in embryos, for instance, introduces complex moral and societal questions, particularly regarding consent and the potential for misuse in non-therapeutic applications. A balanced approach is needed, ensuring that these technologies are developed responsibly and equitably while prioritizing patient safety and ethical considerations [17].

## 8. Conclusions and Future Directions

In conclusion, neonatal diabetes (NDM) is a rare and complex genetic disorder that presents unique challenges in both diagnosis and management. Identifying KCNJ11 and ABCC8 mutations has significantly advanced our understanding of NDM and enabled the development of targeted therapies, particularly sulfonylureas, which have greatly improved glycemic control and reduced the need for insulin injections [9].

However, several challenges remain in the management of NDM, particularly concerning neurological sequelae and the long-term effects of sulfonylureas on neurodevelopment. The persistence of developmental delays, learning disabilities, and epilepsy in some patients suggests that glycemic control alone is not sufficient to address the full spectrum of NDM symptoms [16,17].

## Data Availability

The original contributions presented in the study are included in the article/Supplementary Material, further inquiries can be directed to the corresponding author.

## Supplementary Materials

The following supporting information can be downloaded at: https://www.mdpi.com/article/10.3390/children12020219/s1, Figure S1. Different genetic causes of DMN in patients born to non-consanguineous and consanguineous parents.

## References

[1] Barbetti F., Deeb A., Suzuki S. (2024). Neonatal diabetes mellitus around the world: Update 2024. J. Diabetes Investig..

[2] Beltrand J., Busiah K., Vaivre-Douret L., Fauret A.L., Berdugo M., Cavé H., Polak M. (2020). Neonatal Diabetes Mellitus. Front. Pediatr..

[3] Flanagan S.E., Edghill E.L., Gloyn A.L., Ellard S., Hattersley A.T. (2006). Mutations in KCNJ11, which encodes Kir6.2, are a common cause of diabetes diagnosed in the first 6 months of life, with the phenotype determined by genotype. Diabetologia.

[4] Aguilar-Bryan L., Nichols C.G., Wechsler S.W., Clement J.P., Boyd A.E., González G., Herrera-Sosa H., Nguy K., Bryan J., Nelson D.A. (1995). Cloning of the beta cell high-affinity sulfonylurea receptor: A regulator of insulin secretion. Science.

[5] Greeley S.A.W., Tucker S.E., Naylor R.N., Bell G.I., Philipson L.H. (2010). Neonatal diabetes mellitus: A model for personalized medicine. Trends Endocrinol. Metab..

[6] Grulich-Henn J., Wagner V., Thon A., Schober E., Marg W., Kapellen T.M., Haberland H., Raile K., Ellard S., Flanagan S.E. (2010). Entities and frequency of neonatal diabetes: Data from the diabetes documentation and quality management system (DPV). Diabet. Med..

[7] Pezzotta F., Sarale N., Spacco G., Tantari G., Bertelli E., Bracciolini G., Secco A., D’annunzio G., Maghnie M., Minuto N. (2024). Safety and Efficacy of Using Advanced Hybrid Closed Loop Off-Label in an Infant Diagnosed with Per-manent Neonatal Diabetes Mellitus: A Case Report and a Look to the Future. Children.

[8] Greeley S.A.W., Polak M., Njølstad P.R., Barbetti F., Williams R., Castano L., Raile K., Chi D.V., Habeb A., Hattersley A.T. (2022). ISPAD Clinical Practice Consensus Guidelines 2022: The diagnosis and management of monogenic diabetes in children and adolescents. Pediatr. Diabetes.

[9] Pearson E.R., Flechtner I., Njølstad P.R., Malecki M.T., Flanagan S.E., Larkin B., Ashcroft F.M., Klimes I., Codner E., Iotova V. (2006). Switching from insulin to oral sulfonylureas in patients with diabetes due to Kir6.2 mutations. N. Engl. J. Med..

[10] Rubio-Cabezas O., Ellard S. (2013). Diabetes mellitus in neonates and infants: Genetic heterogeneity, clinical approach to diagnosis, and therapeutic options. Horm. Res. Paediatr..

[11] Lemelman M.B., Letourneau L., Greeley S.A.W. (2018). Neonatal Diabetes Mellitus: An Update on Diagnosis and Management. Clin. Perinatol..

[12] Karges B., Meissner T., Icks A., Kapellen T., Holl R.W. (2011). Management of diabetes mellitus in infants. Nat. Rev. Endocrinol..

[13] Letourneau L.R., Carmody D., Wroblewski K., Denson A.M., Sanyoura M., Naylor R.N., Philipson L.H., Greeley S.A.W. (2017). Diabetes Presentation in Infancy: High Risk of Diabetic Ketoacidosis. Diabetes Care.

[14] De Franco E., Flanagan S.E., Houghton J.A.L., Lango Allen H., Mackay D.J.G., Temple I.K., Ellard S., Hattersley A.T. (2015). The effect of early, comprehensive genomic testing on clinical care in neonatal diabetes: An international cohort study. Lancet.

[15] Hay W.W., Rozance P.J. (2018). Neonatal Hyperglycemia-Causes, Treatments, and Cautions. J. Pediatr..

[16] Carmody D., Pastore A.N., Landmeier K.A., Letourneau L.R., Martin R., Hwang J.L., Naylor R.N., Hunter S.J., Msall M.E., Philipson L.H. (2016). Patients with KCNJ11-related diabetes frequently have neuropsychological impairments compared with sibling controls. Diabet. Med..

[17] Proks P., Arnold A.L., Bruining J., Girard C., Flanagan S.E., Larkin B., Colclough K., Hattersley A.T., Ashcroft F.M., Ellard S. (2006). A heterozygous activating mutation in the sulphonylurea receptor SUR1 (ABCC8) causes neonatal diabetes. Hum. Mol. Genet..

