# Supplementary material for: Diagnosis and Treatment of Neonatal Diabetes Caused by ATP-Channel Mutations: Genetic Insights, Sulfonylurea Therapy, and Future Directions
Source: Children (Basel). 2025 Feb 12;12(2):219. doi: 10.3390/children12020219 (PMC11854417; doi:10.3390/children12020219)
Supplement: Supplementary file 1 [file children-12-00219-s001.zip › children-3179336-supplementary.pdf]

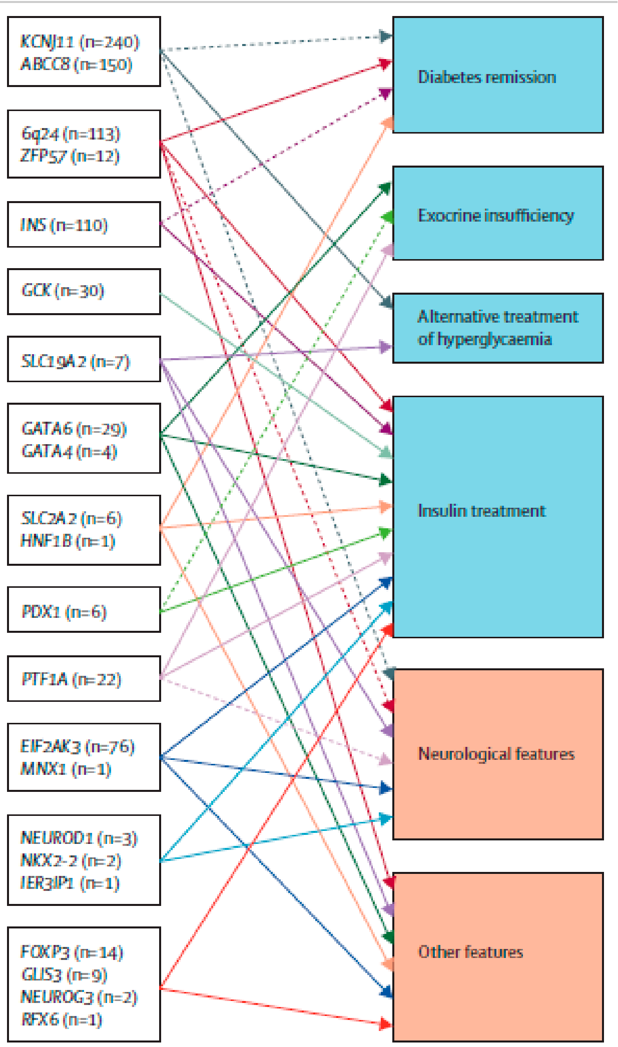

Figure S1: Different genetic causes of DMN in patients born to non-consanguineous and consanguineous parents.
